# Down Modulation of Host Immune Response by Amino Acid Repeats Present in a *Trypanosoma cruzi* Ribosomal Antigen

**DOI:** 10.3389/fmicb.2017.02188

**Published:** 2017-11-10

**Authors:** Carlos A. Toro Acevedo, Bruna M. Valente, Gabriela A. Burle-Caldas, Bruno Galvão-Filho, Helton da C. Santiago, Rosa M. Esteves Arantes, Caroline Junqueira, Ricardo T. Gazzinelli, Ester Roffê, Santuza M. R. Teixeira

**Affiliations:** ^1^Departamento de Bioquímica e Imunologia, Universidade Federal de Minas Gerais, Belo Horizonte, Brazil; ^2^Departamento de Patologia Geral, Universidade Federal de Minas Gerais, Belo Horizonte, Brazil; ^3^Instituto de Pesquisas René Rachou, Fundação Oswaldo Cruz, Belo Horizonte, Brazil

**Keywords:** *Trypanosoma cruzi*, immune response, B-cell, immunomodulation, virulence factors, antigen, repeat domains

## Abstract

Several antigens from *Trypanosoma cruzi*, the causative agent of Chagas disease (CD), contain amino acid repeats identified as targets of the host immune response. Ribosomal proteins containing an Ala, Lys, Pro-rich repeat domain are among the *T. cruzi* antigens that are strongly recognized by antibodies from CD patients. Here we investigated the role of amino acid repeats present in the *T. cruzi* ribosomal protein L7a, by immunizing mice with recombinant versions of the full-length protein (TcRpL7a), as well as with truncated versions containing only the repetitive (TcRpL7aRep) or the non-repetitive domains (TcRpL7aΔRep). Mice immunized with full-length TcRpL7a produced high levels of IgG antibodies against the complete protein as well as against the repeat domain, whereas mice immunized with TcRpL7aΔRep or TcRpL7aRep produced very low levels or did not produce IgG antibodies against this antigen. Also in contrast to mice immunized with the full-length TcRpL7a, which produced high levels of IFN-γ, only low levels of IFN-γ or no IFN-γ were detected in cultures of splenocytes derived from mice immunized with truncated versions of the protein. After challenging with trypomastigotes, mice immunized with the TcRpL7a were partially protected against the infection whereas immunization with TcRpL7aΔRep did not alter parasitemia levels compared to controls. Strikingly, mice immunized with TcRpL7aRep displayed an exacerbated parasitemia compared to the other groups and 100% mortality after infection. Analyses of antibody production in mice that were immunized with TcRpL7aRep prior to infection showed a reduced humoral response to parasite antigens as well as against an heterologous antigen. *In vitro* proliferation assays with mice splenocytes incubated with different mitogens in the presence of TcRpL7aRep resulted in a drastic inhibition of B-cell proliferation and antibody production. Taken together, these results indicate that the repeat domain of TcRpL7a acts as an immunosuppressive factor that down regulates the host B-cell response against parasite antigens favoring parasite multiplication in the mammalian host.

## Introduction

Chagas disease (CD) or American trypanosomiasis, caused by the blood-borne parasite *Trypanosoma cruzi*, is a tropical neglected disease that currently affects about 10 million people in Latin America^1^. The infection begins with an acute phase characterized by high parasite numbers in the bloodstream, which, in spite of that, is usually asymptomatic. During the chronic phase, which usually begins 2–3 months after infection, parasitemia is undetectable, two distinct clinical forms can occur; an indeterminate, without symptoms, and a symptomatic form of CD which affects approximately 30% of patients, in which hidden parasites, mainly inside heart and digestive muscle cells can cause serious cardiac and/or digestives alterations (Prata, 2001; Teixeira et al., 2006; Junqueira et al., 2010; Rassi and de Rezende, 2012). The two drugs that are currently available for treatment of CD, Nifurtimox and Benzonidazol, thought to be effective only in acute phase, have several side effects (Castro et al., 2006). The parasite is transmitted to humans through the bite of a reduvidae insect vector known as “kissing bug.” Although efforts toward the control of vector transmission have had a positive impact in several Latin America countries (Coura et al., 2014), development of a prophylactic vaccine remains a priority if we wish to prevent novel cases of CD in areas where infected vectors and mammalian reservoirs are still easily found.

Although a complex immune response known to involve immunoregulatory mechanisms that are associated with the pathology of CD has been thoroughly investigated, it is still far from being fully understood. Studies in experimental models of *T. cruzi* infection have shown the essential role of cytotoxic CD8^+^-specific cells as well as of a strong humoral immune response against parasite antigens (Tarleton, 2007; Dumonteil, 2009; Rodrigues et al., 2012; Vasconcelos et al., 2014). Significant efforts toward the development of an effective vaccine against this parasite have been made using various antigens, such as cruzipain (Cazorla et al., 2010), amastigote surface protein (Nogueira et al., 2013), paraflagellar rod protein (Kurup and Tarleton, 2014), and different members of the *trans*-sialidase (TS) surface protein family (Hoft et al., 2007; Rosenberg et al., 2010) as well as using different immunization protocols that promote a Th1 response with stimulation of CD8^+^ T cells (Pereira et al., 2015). Yet, none of these vaccine candidates has proven completely effective, probably due to the complex mechanisms that the parasite develops to escape from the host immune response.

Intensive efforts to search for *T. cruzi* antigens to be employed as targets for serodiagnosis as well as vaccine components resulted in the identification of a large number of proteins containing repeated amino acid sequences. *In silico* analyses based on the complete genome sequences of several pathogens and non-pathogenic microorganisms showed that the predicted proteome of intracellular protozoan parasites has a higher repetitive content than the proteome from extracellular parasites and free-living protists (Fankhauser et al., 2007; Mendes et al., 2013). Indeed, tandemly repeated amino acid sequences, which have been implicated with binding to host receptors as well as with immune-evasion mechanisms, are present in many surface proteins of intracellular protozoan parasites such as *Plasmodium* spp., *Leishmania* spp., and *T. cruzi*. Several *Leishmania* and *T. cruzi* proteins containing repeated amino acid motifs have been described as targets of B-cell immune response and a bias toward the expression of these proteins in the amastigote stage further suggests their involvement with intracellular parasitism (Goto et al., 2008, 2010). The *Leishmania* surface proteins A2 (Fernandes et al., 2014), HASP (Depledge et al., 2010), and PSA (Boceta et al., 2000), all of them containing large repeat domains, have been identified as antigens that are strongly recognized by antibodies from infected individuals. Among the *T. cruzi* surface proteins containing tandemly repeated amino acids known to be targets of the host immune response are B13 antigen (Duranti et al., 2012), TSs (Freitas et al., 2011), mucins (Giorgi and de Lederkremer, 2011), and the mucin-associated surface protein (MASP) (dos Santos et al., 2012). A sub-group of the TS protein family, which is encoded by the largest *T. cruzi* gene family, with more than 1,000 copies in the parasite genome (El-Sayed et al., 2005; Dc-Rubin and Schenkman, 2012), contains at its C-terminal region a amino acid repeat domain known as shed acute phase antigen (SAPA). Several studies suggested that the SAPA repeat is a T-cell independent antigen type I, which is a B-cell mitogen that acts as a diversion for the immune system, luring away antibodies from the TS catalytic site (Risso et al., 2007; Dc-Rubin and Schenkman, 2012).

Parasite ribosomal proteins containing amino acids repeats are also among highly immunogenic antigens identified in studies in which sera from individuals with leishmaniasis and CD were used (Ramírez et al., 2013; Longhi et al., 2014). One such antigen is the *T. cruzi* homolog of the eukaryotic ribosomal protein L7a, a component of the large subunit of the ribosome. We have previously shown that the Ala–Lys–Pro-rich repeat domain present at the N-terminus of the *T. cruzi* L7a is a target for antibodies from CD patients and that the number of repeats varies among different parasite strains (Cerqueira et al., 2008; Pais et al., 2008). Here, we investigated the role of the amino acid repeats present in *T. cruzi* L7a protein by generating recombinant versions of the complete antigen (TcRpL7a) as well as truncated versions containing only its repetitive (TcRpL7aRep) or the non-repetitive domain (TcRpL7aΔRep) and using the three antigens to immunize mice. After evaluating the immune response elicited in mice immunized which each antigen we asked which version of the TcRpL7a protein is able to induce protection against a challenge with a virulent *T. cruzi* strain. The result of immunization experiments together with the results of *in vitro* proliferation assays indicated that the repeat domain on *T. cruzi* L7a has immune regulatory properties that help the parasite to evade the specific host immune response.

## Materials and Methods

### Mice, Parasites, and Sera Samples

All experiments were done with 6- to 8-week-old BALB/c female mice, purchased from the Centro de Bioterismo Central from the Universidade Federal de Minas Gerais. The design and methodology of all experiments involving mice were performed in accordance with the guidelines of COBEA (Brazilian College of Animal Experimentation), strictly followed the Brazilian law for “Procedures for the Scientific Use of Animals” (11.794/2008) and were approved by the animal-care ethics committee of the Federal University of Minas Gerais (CEUA, UFMG) under the protocol number 133/2014. The protocol to obtain human sera used in this study was approved by the Human Ethics Committee of the Federal University of Minas Gerais (COEP, UFMG) (protocol number 312/06). Blood form trypomastigotes (BFT) and cell culture trypomastigotes (TCT) of the CL Brener clone of *T. cruzi* were used. The BFT were maintained every 2 weeks by intraperitoneal (i.p.) inoculation of BALB/c mice with blood from infected mice. TCTs were maintained by infection of confluent LLC-MK2 cell line, every week, maintained at 37°C with 5% CO_2_, in DMEM medium (Gibco) with 10% of fetal bovine serum (FBS). Homogenate of *T. cruzi* extracts (TcExtract) was prepared from trypomastigotes released from cell culture infection as follows. Supernatant from LLC-MK2 infected cells was harvested 7–8 days after infection and centrifugated at 2,000 × *g* for 5 min to remove culture cells. Next, the supernatant was centrifugated at 10,000 × *g* for 10 min and trypomastigotes in pellet were washed with phosphate-buffered saline (PBS) three times. Parasites were thermic shock-lysed by 10 cycles of freezing and thawing. The homogenate was suspended in sterile PBS and the protein amount was quantified by Bradford assay (BioRad).

### Bioinformatics Analyses

Sequences retrieved from Tritrypdb^2^ or GenBank were aligned using Multiple Sequence Comparison by Log-Expectation (MUSCLE). Predictions for B cells linear epitopes were carried out using BepiPred server 1.0 with a threshold of 0.50 (Larsen et al., 2006). For T-cell epitopes prediction we employed tools from the Immune epitope database (IEDB) which uses a combination of methods to predict best peptides for MHC II and I (Wang et al., 2008). Results are expressed in percentile scores and top 0.5% was selected as binders.

### Plasmid Constructions and Production of Recombinant Proteins

The coding sequence for TcRpL7a (GenBank accession number AF316150) was amplified from *T. cruzi* genomic DNA purified from the CL Brener clone using the following primers 5′-CCGCTAGCCCCGGCAAGGAAGTGAAA-3′ and 5′-ATGTCGACCATTACGGCGGCAGCATC-3′ containing sites for *Nhe* I and *Sal* I restriction enzymes, respectively. A DNA fragment encoding the N-terminal 90 amino acids of TcRpL7a (TcRpL7aRep) was amplified using 5′-CCGCTAGCCCCGGCAAGGAAGTGAAA-3′ and 5′-GGCTCGAGCACAAAAGGTGAGATGGC-3′ primers containing sites for *Nhe* I and *Xho* I restriction enzymes, respectively. A DNA fragment encoding the C-terminal 227 amino acids for TcRpL7a (TcRpL7aΔRep) was amplified using 5′-GGGCTAGCATCTCACCTTTTGTGGCGC-3′ and 5′-ATGTCGACCATTACGGCGGCAGCATC-3′ primers containing *Nhe* I and *Sal* I sites, respectively. All three PCR amplified DNA products containing TcRpL7a, TcRpL7aRep, and TcRpL7aΔRep sequences were digested with compatible enzymes and cloned into pET21 (Novagen) expression vector to generate his-tagged proteins. Bacteria BL21 Star^TM^ (DE3) was used for transformation following the protocols provided by the manufacturer. After IPTG induction, recombinants histidine-tagged proteins were purified by Ni^2+^ chromatography with HisTrap column following the protocols provided by the column supplier (GE HealthCare). Recombinant proteins TcRpL7a and TcRpL7aΔRep were obtained as inclusion bodies and solubilized with 4 and 8 M urea, respectively, before applying to the Ni^2+^ column. After purification, all proteins were dialyzed against PBS. LPS was removed using Triton X-114 and LPS contamination in the proteins was determined by limulus amebocyte lysate (LAL) assay kit following the manufacturer protocol (Alka do Brasil).

### Immunization, Challenge, and Cytokine Measurement

Three groups of 10 BALB/c female mice received prime-boost-boost immunizations by subcutaneous (s.c) injections of 10 μg of each of the three different recombinant proteins plus 18 μg of CpG B344 (Alpha DNA) and 30% (v/v) of alum rehydragel L.V. solution (Reheis), in a volume of 100 μl of saline solution, with an interval of 14 days. The fourth group of mice received injections with PBS plus CpG and alum. Nine days after the last boost, blood samples were collected from four animals in each group. The blood was centrifuged for 10 min at 2,700 × *g*, the sera collected, and stored at -20°C. To evaluate the cellular immune response four animals were euthanized 3 weeks after the last boost and the spleens were collected. Spleen mouse cells (SMCs) were cultured by harvesting spleen and smashing through 100 μm pore cell Strainer (Cell Strainer BD Falcon) and treated with ACK buffer (NH_4_Cl 0.15 M, KHCO_3_ 0.1 M e Na_2_EDTA 0.1 M) for erythrocytes lysis, then washed twice with RPMI medium 1640 (Gibco), supplemented (complete RPMI medium) with 10% FBS (Gibco), 2 mM glutamine, 100 μg/ml streptomycin, and 100 U/ml penicillin. The viability of the cells was evaluated by using 0.2% trypan blue to discriminate between live and dead cells. 1 × 10^6^ SMC/ml was incubated for 72 h at 37°C in 24-well plates with complete RPMI medium in the presence of 10 μg/μl of each recombinant protein. Cytokines (IFN-γ and IL-10) were measured in supernatants using R&D Systems Kits by enzyme-linked immunosorbent assays (ELISAs), following manufacturer instructions. The remaining animals were challenged with 5,000 BFT of the CL Brener clone. Parasitemia, determined by direct microscopy examination the number of parasites in 5 μl of blood taken from the animal tail, was monitored three times a week. Heart tissues were collected from the animals at the peak of the parasitemia for histopathological analyses and analyzed as described in the following sessions. Sera were also collected from both groups of mice at the pick of parasitemia to determine antibody levels against total parasite extracts as indicated before.

### Histopathological Analyses

Infected and TcRpL7aRep immunized/infected mice were sacrificed, the hearts were harvested, and fixed in 4% buffered paraformaldehyde. Fixed hearts were embedded in paraffin and sliced into 7 μm sections by microtome and stained with hematoxylin and eosin (H&E). Twenty fields from one heart tissue section per mice were analyzed by microscopy under 40× magnification. The numbers of intact amastigote nests and the percentages of inflammatory cellular infiltrate were determined using the ImageJ Software.

### Western Blot and ELISA

For western blot analyses, proteins were first separated by SDS–PAGE, by using a 5% stacking gel and 12.5% resolving gel. Then, the proteins were transferred to nitrocellulose membranes, that were blocked in PBS-T (PBS plus 0.01% Tween 20) containing 5% of low fat milk for 1 h following by a overnight incubation at room temperature with primary antibodies diluted in PBS-T containing 1% of low fat milk. The membranes were washed and incubated with peroxidase-conjugated secondary antibodies (SoutherBiotech), diluted in PBS-T containing 1% of low fat milk, and submitted to chemoluminescent reaction using Luminata reagent (Millipore). For histidine tag detection, mouse anti-his monoclonal antibodies (GE Healthcare) were used in a 1:2,000 dilution. For specific antibody response, as primary antibodies were used a pool of sera from 10 chronic CD patients in a 1:250 dilution, or sera from mice acutely or chronically infected with *T. cruzi* in a 1:200 dilution. Serum from immunized mice, in a dilution of 1:200 also were used as primary antibodies. Secondary anti-human IgG antibodies (SoutherBiotech) was diluted 1:1,000 and secondary anti-mouse IgM or IgG antibodies (SoutherBiotech) were diluted 1:2,000. Sera from immunized or infected mice were analyzed by ELISA. Briefly, 96 wells microtiter plates (Nunc Immunoplates) were coated with TcRpL7a, TcRpL7aRep, and TcRpL7aΔRep recombinant proteins or with *T. cruzi* extract (diluted all of them at 10 μg/ml in 100 μl of carbonate buffer per well, pH 9.6) and incubating at 4°C overnight. Mice sera were diluted 1:100 in blocking buffer (PBS, 0.05% Tween 20, and 3% low fat milk) and incubated for 1 h at 37°C. Plates were incubated with peroxidase-conjugated goat anti-mouse IgM, IgG, IgG1, and IgG2a (SouthernBiotech) 1 h at 37°C, and peroxidase reactions were detected by incubation with 3,3′,5,5′-tetramethylbenzidine (TMB 1 mg/ml) reagent (SIGMA) at room temperature and optic density was measured spectrophotometrically at 450 nm. To determine the levels of non-specific IgM antibodies, plates were coated as described before with 100 μl of a 1/1,000 dilution of anti-mouse unlabeled α-IgM (SoutherBiotech) and 1:100 dilution of supernatants of cells cultures were added followed by the addition of peroxidase-conjugated anti-mouse α-IgM as described before.

### Hypersensitivity Response against Ovalbumin

Two group of healthy BALB/c mice, five mice per group, were immunized s.c. with 100 μg of Ovalbumin (OVA; Sigma–Aldrich) diluted in 20 μl of saline buffer and emulsified with 30 μl of complete Freud’s adjuvant (CFA; BD BioSciences). The OVA antigen was given twice with 10 days interval, and one group of mice was immunized with 10 μg TcRpL7aRep diluted in 100 μl of saline buffer, 24 h before each OVA immunization. Sera were collected at days 7 and 17 after the first immunization, and anti-OVA antibodies were determined by ELISA using plates coated with 1 μg of OVA antigen/well as described before. Hypersensitivity response was elicited in both group of mice by injecting 30 μl of heat aggregated OVA (HAO) in one rear footpad and saline buffer in the other. Footpad swelling was measured 24, 48, and 72 h after HAO injection and was compared with the basal footpad thickness. HAO was prepared by heating a 2% OVA solution in PBS at 70°C for 1 h. After cooling slowly at room temperature, the antigen was centrifuged at 225 × *g* for 10 min at 4°C, then was washed twice with PBS and resuspended to the original volume in PBS, as described elsewhere (Titus and Chiller, 1981).

### *In Vitro* Stimulation and Flow Cytometry Analyses of Splenocytes

Two healthy 5- to 6-week-old BALB/c female mice were sacrificed and the spleens were harvested and processed for erythrocyte lysis as described before. Adherent cells were removed by incubating disrupted spleen tissue in 24 wells plated overnight at 37°C in complete RPMI medium. Next day, all non-adherent splenocytes were taken and the total number of viable cells was determined by Trypan blue exclusion. A total of 50 × 10^6^ splenocytes were centrifuged at 2,000 × *g* for 5 min, and the pellet was resuspended in 1 ml of PBS with 5% of FBS. The cell suspension was mixed with 110 μl of 50 μM carboxyfluorescein succinimidyl ester (CFSE; Sigma–Aldrich) prepared in PBS and incubated for 5 min at room temperature, in the darkness. Cells were washed three times with 10 volumes of PBS with 5% FBS and resuspended in complete RPMI medium. CFSE labeled spleen cells were incubated at 37°C for 24 h, at a cell density of 10,000 cells/μl in complete RPMI medium (Control) or in the presence of 10 μg/ml of TcRpL7aRep. After this incubation period, the cells were stimulated with RPMI medium (Control), concanavalin A (ConA; Sigma–Aldrich) at 5 μg/ml, lipopolysaccharide (LPS; Sigma–Aldrich) at 10 μg/ml or by adding to plates that were previously coated with hamster anti-mouse CD3 IgG (BD BioSciences). To coat wells with α-CD3, 50 μl of a 5 ng/ml solution of anti-CD3 diluted in sterile PBS 1× was added to the wells and incubated at 4°C overnight. CFSE labeled spleen cells were also stimulated with ConA, LPS, or anti-CD3 in the presence of 10 μg/ml of TcRpL7aRep. Seventy-two or 120 h after stimulation cells were centrifuged at 2,000 × *g* for 5 min, washed with PBS with 2% FBS, and incubated for 1 h with Fc Block (BD BioSciences clone 2.4G2). Cells were washed with PBS, centrifuged at 2,000 × *g* for 5 min, and resuspended with 50 μl of different anti-mouse monoclonal antibodies solutions, and incubated for 30 min at 25°C. The following fluorescent monoclonal antibodies were used: anti-CD19-APC-eFluor450 (eBioscience, clone 1D3), anti-CD3-Fluor450 (eBioscience, clone 17A2), anti-CD4-AlexaFluor700 (eBioscience, clone GK1.5), anti-CD8-PE-Cy5 (BD, clone 53-6.7), and anti-CD25-PE (Biolegend, clone 3C7). After staining with each fluorescent antibody, cells were washed, resuspended in PBS, and analyzed in a LSRFortessa^TM^ flow cytometer (BD BioSciences). Data were analyzed using the FlowJo V10 software.

### Statistical Analysis

Data were analyzed using GraphPad Prism version 5.0 software and results presented as means ± SD. Statistical significance was performed using one-way ANOVA and non-parametric test followed by Bonferroni post-test. *P* < 0.05 was considered statistically significant.

## Results

### L7a *T. cruzi* Ribosomal Protein Contains Amino Acid Repeats Not Found in Any Other Eukaryotic L7a

**FIGURE 1:**
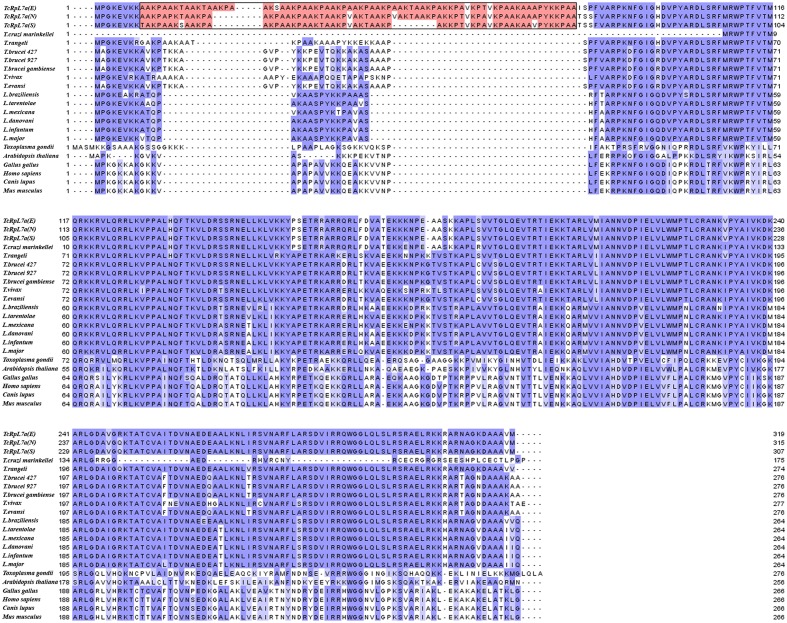
*Trypanosoma cruzi* L7a contains amino acid repeats in its N-terminus that are absent in homologous proteins from other eukaryotes. Amino acid sequence of *T. cruzi* L7a derived from the two alleles present in the CL Brener genome [TcRpL7a(E), TcCLB.506401.320 – Esmeraldo like and TcRpL7a (N), TcCLB.510835.40 – non-Esmeraldo] and the gene present in the SylvioX-10 clone [TcRpL7a(S), TCSYLVIO_005433] were aligned and compared to L7a amino acid sequences from *T. cruzi marinkellei* (Tc_MARK_7171), *T. rangeli* (TRSC58_03561), two *T. brucei* strains (Tb427.08.1330 and Tb927.8.1330), *T. brucei gambiensis* (Tbg972.8.880), *T. vivax* (TvY486_0800730), *T. evansi* (TevSTIB805.8.1230), six *Leishmania* species (*L. brasiliensis*, LbrM.07.0560; *L. tarentoale*, LtaPcontig477-1; *L. mexicana*, LmxM.07.0500; *L. donovani*, LdBPK_070550.1; *L. infantum*, LinJ.07.0550; and *L. major*, LmjF.07.0500), *Toxoplasma gondii* (EPT25904.1) as well as from five other eukaryotes such as chicken (RL7A_CHICK P32429), dog (NM_001286939.1), mouse (NM_013721.3), plant (AL162651.1), and human (NP_000963.1). The region containing the repetitive motif AAKX, present only in *T. cruzi* L7a, is indicated by the rectangle.

We have previously shown that ribosomal proteins containing amino acid repeats are important targets of the humoral immune response of patients with CD (DaRocha et al., 2002). Immunoscreening of a *T. cruzi* cDNA library showed that two clones encoding sequences homologous to ribosomal proteins containing similar amino acid repeats, named TcRpL7a and TcRpL19, are among the cDNA clones recognized by antibodies from CD patients (DaRocha et al., 2002). Sequence analyses of the *T. cruzi* genome database also showed that a total of six *T. cruzi* sequences homologous to eukaryotic ribosomal proteins contain amino acid repeats in their sequences (Pais et al., 2008). The alignment of the complete amino acid sequences of L7a genes present in the genomes of different *T. cruzi* strains with amino acid sequences from L7a of several other eukaryotes, including the related protozoan parasites *Trypanosoma brucei*, *Trypanosoma rangeli*, and *Leishmania* spp., showed that only *T. cruzi* L7a has a repetitive amino acid domain at its N-terminus (**Figure 1**). Besides being highly conserved in different *T. cruzi* strains, a similar Ala, Lys, Pro-rich repeated motif is also found in *T. cruzi* homologs of L7a, L19, and L23a ribosomal proteins (Cerqueira et al., 2008; Pais et al., 2008). It is noteworthy that, in spite of being a highly conserved protein, L7a sequences from members of the Trypanosomatidae family such as *T. rangeli*, *T. brucei*, *T. vivax*, *T. cruzi marinkellei*, *T. evansi*, and several *Leishmania* species do not present a repeat domain.

**FIGURE 2:**
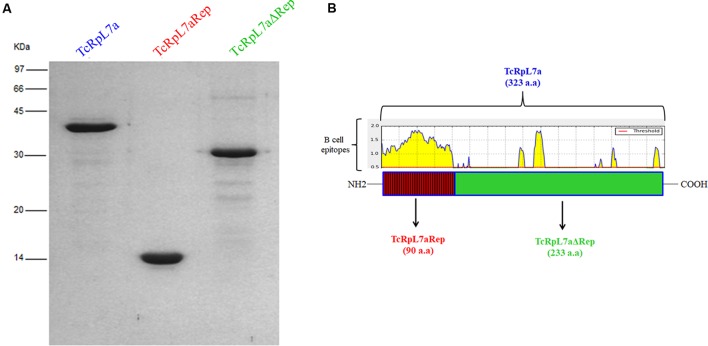
Purified recombinant versions of TcRpL7a and B-cell epitope localization. SDS–PAGE of his-tagged recombinant full-length TcRpL7a protein and its truncated versions, TcRpL7aRep and TcRpL7aΔRep, purified by Ni-affinity chromatography **(A)**. Schematic representation of the aminoacid sequence of the TcRpL7a protein with its repetitive domain (TcRpL7aRep, in red) at the N-terminal region, and the non-repetitive domain (TcRpL7aΔRep, in green) at the C-terminal region; the localization of linear B-cell epitope prediction shown in yellow was performed with BepiPred with a threshold of 0.5 **(B)**.

We have also previously shown that recombinant TcRpL7a in fusion with glutathione *S*-transferase (GST) or a synthetic peptide containing five TcRpL7a repeated motifs may be employed as targets in serological tests since they are strongly recognized by antibodies present in sera from patients with CD but not from patients with other parasitic diseases (Pais et al., 2008). To investigate the role of TcRpL7a repeats during the immune response elicited after infection by *T. cruzi*, we produced three recombinant version of the TcRpL7a protein: one containing only the 90 amino acid N-terminal region corresponding to the repeat domain (TcRpL7aRep, 10 kDa), the second containing a 223 amino acid C-terminal region, corresponding to the non-repetitive domain (TcRpL7aΔRep, 30 kDa), and a third version containing the complete, 319 amino acid, *T. cruzi* L7a antigen (TcRpL7a, 45 kDa). All recombinant antigens contain a histidine tag and were purified by affinity chromatography with Ni^+2^ columns (**Figure 2A**).

B-cell epitope prediction analysis by BepiPred software indicated the presence of eight linear epitopes in TcRpL7a sequence (**Figure 2B**, **Supplementary Figure S1A** and **Supplementary Table S1**) with scores higher than 0.5. However, the most relevant epitopes, scoring higher than 1.0, correspond to the complete repetitive region and one 15 mer sequence in the non-repetitive region of the protein (**Supplementary Figure S1A** and **Supplementary Table S1**). On the other hand, T-cell epitope prediction by IEDB found seven epitopes with percentile ranks below 0.5% for MHC class I, all of them, but one, located in the non-repetitive portion of the protein (**Supplementary Figure S1B** and **Supplementary Table S1**). The best binder, an 8 mer sequence with 0.1 percentile score, is predicted to bind strongly to two BALB/c MHC I molecules, H-2-Ld and H-2-Dd. For MHC II, only one peptide was found within rank percentile below 0.5% predicted to bind H2-IAd (BALB/c MHC II) (**Supplementary Table S1**) and this peptide is located in the non-repetitive region of the protein (**Supplementary Figure S1C**). Therefore, while the repetitive region of TcRpL7a seems to concentrate most B-cell epitopes, T-cell epitopes were mostly present at the non-repetitive region.

**FIGURE 3:**
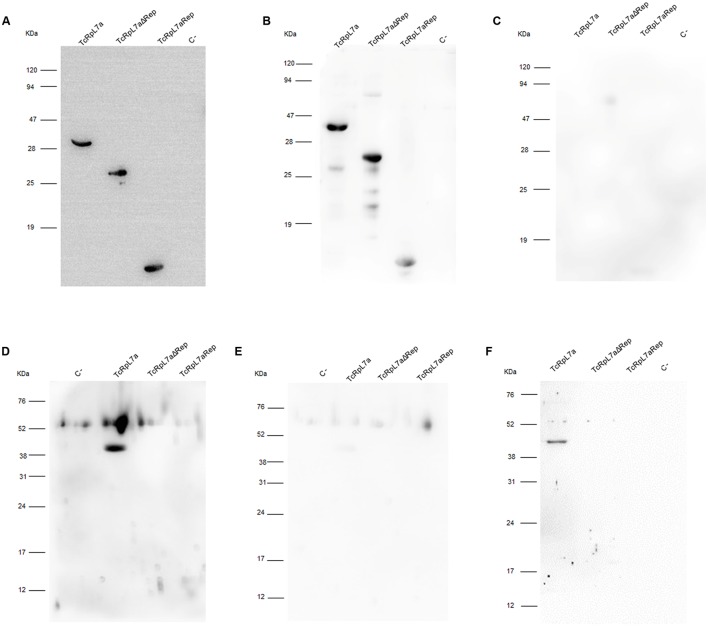
Distinct domains of TcRpL7a are recognized by antibodies present in sera from patients with CD and from *T. cruzi-*infected mice. Western blot analyses of purified recombinant proteins TcRpL7a, TcRpL7aRep, and TcRpL7aΔRep as well as BSA, used as a negative control (C-) probed with anti-his antibody **(A)**; a pool of sera from 10 CD patients **(B)**; a pool of sera from 10 uninfected individuals **(C)**; a pool of sera from BALB/c mice acutely infected with *T. cruzi* CL Brener strain, 14 days post-infection **(D,F)**; a pool of sera from uninfected mice **(E)**. Following primary antibody incubations, membranes were incubated with horseradish peroxidase (HRP)-conjugated anti-mouse IgG **(A,F)**, HRP-conjugated anti-human IgG **(B,C)**, and HRP-conjugated anti-mouse IgM **(D,E)**. Whereas chronic CD patients have IgG antibodies that recognize all three versions of the recombinant antigen, acutelly infected mice have high levels of IgM antibodies that recognize the full-length antigen.

Using serum pools from individuals chronically infected with *T. cruzi* (**Figure 3B**) and from non-infected controls (**Figure 3C**), we showed that patients with CD produced IgG antibodies against the complete TcRpL7a as well as against the two truncated versions of the recombinant antigen, whereas sera from non-infected individuals have no IgG antibodies that recognize these proteins (**Figure 3C**). In contrast, BALB/c mice acutely infected with *T. cruzi* generated IgM antibodies that strongly recognize only the full-length recombinant protein (**Figure 3D**). As expected, non-infected mice do not produce IgM antibodies that recognize these antigens (**Figure 3E**) whereas mice acutely infected with *T. cruzi* produce IgG antibodies against the full-length protein (**Figure 3F**). All recombinant antigens tested positive in western blots with anti-his tag antibodies (**Figure 3A**).

### Cellular and Humoral Immune Response in Mice Immunized with Different Domains of the TcRpL7a Antigen

**FIGURE 4:**
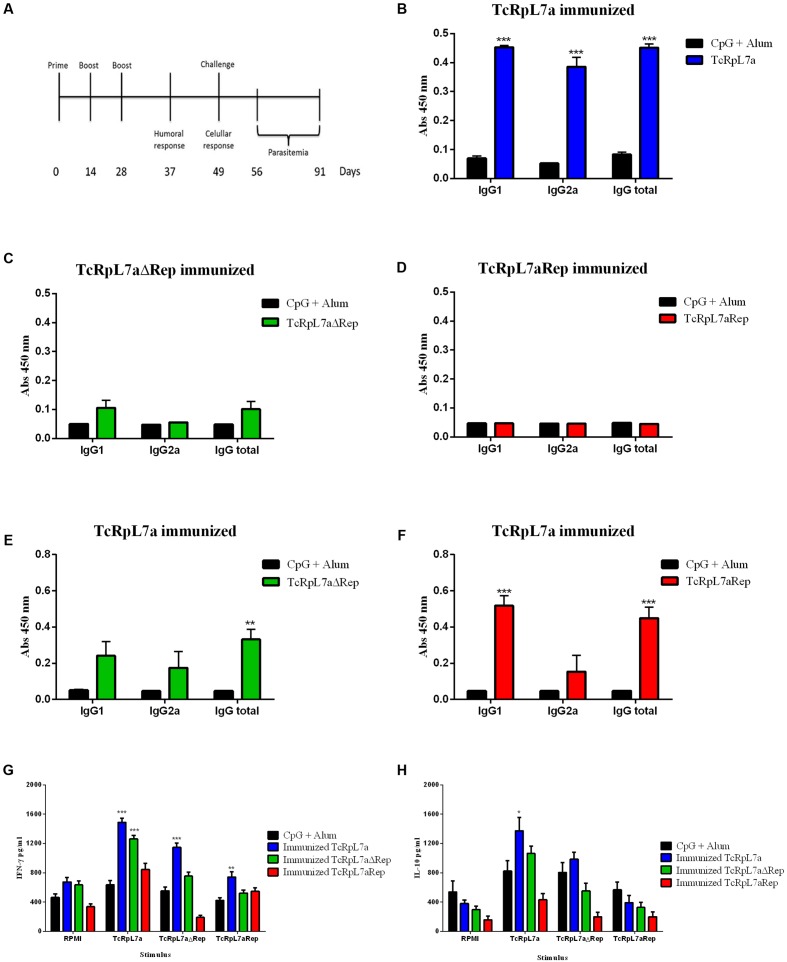
Humoral immune response and celular activation in mice immunized with different versions of recombinantTcRpL7a. Ten BALB/c mice were immunized three times with 10 μg of recombinant proteins plus CpG and alum with an interval of 14 days. Nine days after the last immunization, sera were collected and the humoral responses evaluated. Three weeks after the last immunization, four animals in each group were sacrificed and their spleens were removed and stimulated to determine the cellular immune response. The remaining animals were challenged with 5,000 bloodstream trypomastigotes of CL Brener strain and during 35 days post-infection, parasitemia was evaluated according to the scheme shown in **(A)**. Sera levels of IgG1, IgG2a, and total IgG from mice immunized with each recombinant antigen or from control animals (CpG + Alum) were determined by ELISA using recombinant full-length TcRpL7a protein **(B)**, TcRpL7aΔRep **(C)**, or TcRpL7aRep **(D)** to coat the plates. Levels of IgG1, IgG2a, and total IgG present in sera from mice immunized with full-length TcRpL7a protein were also determined by ELISA using TcRpL7aΔRep **(E)** or TcRpL7aRep **(F)** to coat the plates. For cellular response, splenocytes from TcRpL7a-, TcRpL7aRep-, or TcRpL7aΔRep-immunized mice were incubated for 72 h in presence of one of the three versions of the antigen or in the presence of RPMI medium as a negative control. Levels of IFN-γ **(G)** and IL-10 **(H)** present in the culture supernatants were evaluated by ELISA (^∗∗∗^*P* < 0.001; ^∗∗^*P* < 0.01; ^∗^*P* < 0.05).

To investigate the immune response against the *T. cruzi* L7a protein in a susceptible animal model of infection, we immunized BALB/c mice according to the protocol shown in **Figure 4A**, with all three distinct versions of purified recombinant TcRpL7a antigens (**Figure 2A**). Nine days after the last immunization, specific IgG antibodies present in sera from immunized animals were measured by ELISA. As shown in **Figure 4B**, immunization with the full-length protein induces production of IgG antibodies against TcRpL7a (subtypes IgG1 and IgG2a). In contrast, mice immunized with TcRpL7aΔRep (**Figure 4C**) or with TcRpL7aRep (**Figure 4D**) did not produce significant levels of IgG antibodies against these antigens. Interestingly, immunization with the full-length version results in the production of significant levels of IgG antibodies against both TcRpL7aΔRep and TcRpL7aRep (**Figures 4E,F**). Hence, similar to the humoral immune response observed in CD patients, mice immunized with the full-length TcRpL7a antigen generate a strong humoral immune response against this protein. In contrast, mice immunized with truncated versions of the TcRpL7a antigen generated only a weak or did not elicit a detectable humoral response to the non-repetitive and the repetitive domain, respectively.

Since immunization of BALB/c mice with the truncated versions of the TcRpL7a failed to induce a humoral immune response, we investigated the cellular immune response elicited by the immunization with the different versions of the recombinant antigen. Spleen cells from mice immunized with the full-length antigen as well as with TcRpL7aΔRep and TcRpL7aRep were collected 21 days after the last immunizations and the cellular immune response was accessed by measuring IFN-γ and IL-10 in culture supernatants of splenocytes that were re-stimulated with each of the three recombinant antigens. As shown in **Figure 4G**, splenocytes from animals immunized with the full-length TcRpL7a produced high levels of IFN-γ when they were re-stimulated with the complete protein as well as with the truncated version of TcRpL7a without the repeats. Re-stimulation with the repeat domain or with the full-length antigen did not induce IFN-γ in splenocytes from animals immunized with the repeats (**Figure 4G**). However, splenocytes from animals immunized with the non-repetitive domain of TcRpL7a were able to produce IFN-γ when re-stimulated with the complete antigen. Thus, in contrast to splenocytes from animals immunized with the full-length protein or with the non-repetitive domain, splenocytes from mice immunized with the repetitive domain completely failed to produce IFN-γ when re-stimulated with the truncated version of the antigen (**Figure 4G**). Similarly, only splenocytes from mice immunized with the full-length antigen (TcRpL7a) were able to produce IL-10 after being re-stimulated with the full-length antigen (**Figure 4H**). However, animals that were immunized with the truncated version containing the non-repetitive domain or with antigen containing only the repeat domain did not produce IL-10 at levels that are statistically different from the levels detected in cells from control animals that were stimulated with the different antigens (**Figure 4H**).

### Immunization with Full-Length TcRpL7a Resulted in Partial Protection against a Challenge Infection Whereas Immunization with the Repeat Domain Exacerbates the Infection

**FIGURE 5:**
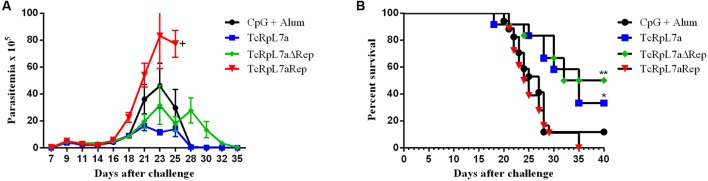
Parasitemia and survival rates after challenging of immunized animals with *T. cruzi*. Four groups of mice were immunized with the three different versions of TcRpL7a recombinant protein or injected only with the adjuvants. Twenty-one days after the last immunization, they were challenged with 5,000 bloodstream trypomastigotes of the CL Brener strain. Parasitemia was followed for 35 days **(A)** and mortality rates **(B)** were determined in each group of mice (^∗∗^*P* < 0.01; ^∗^*P* < 0.05).

Because of the strong differences observed with the immune response in mice immunized with different versions of TcRpL7a, we investigated the outcome of the infection in these animals after challenge with infective *T. cruzi* trypomastigotes. Twenty-one days after the last immunization, we challenged six animals from each group with 5,000 bloodstream trypomastigotes of the CL Brener clone and the parasitemia was monitored for 35 days, beginning 7 days after the challenge. As shown in **Figure 5A**, immunization with the full-length antigen conferred partial protection whereas immunization with TcRpL7aΔRep did not significantly alter parasitemia levels compared to control animals. A 75% reduction in parasitemia in mice immunized with the full-length antigen observed between 3 and 4 weeks after infection was corroborated by the data showing a 60% animal survival 35 days after infection compared to 30% survival of control animals. In contrast, immunization with the antigen containing only the repeated domain resulted in a twofold increase in the levels of parasitemia on day 23 compared to the control groups and eightfold increase in parasitemia compared to animals immunized with the full-length protein (**Figure 5A**). Strikingly, immunization with the repeat domain resulted in 100% mortality with a median survival of 24.5 days showing no level of protection when compared to non-vaccinated animals that showed a median survival of 27 days (**Figure 5B**). In contrast, animals vaccinated with the complete protein or the non-repetitive domain showed increased survival (**Figure 5B**).

### Immunization with TcRpL7aRep Down Regulates the Humoral Immune Response against *T. cruzi*

**FIGURE 6:**
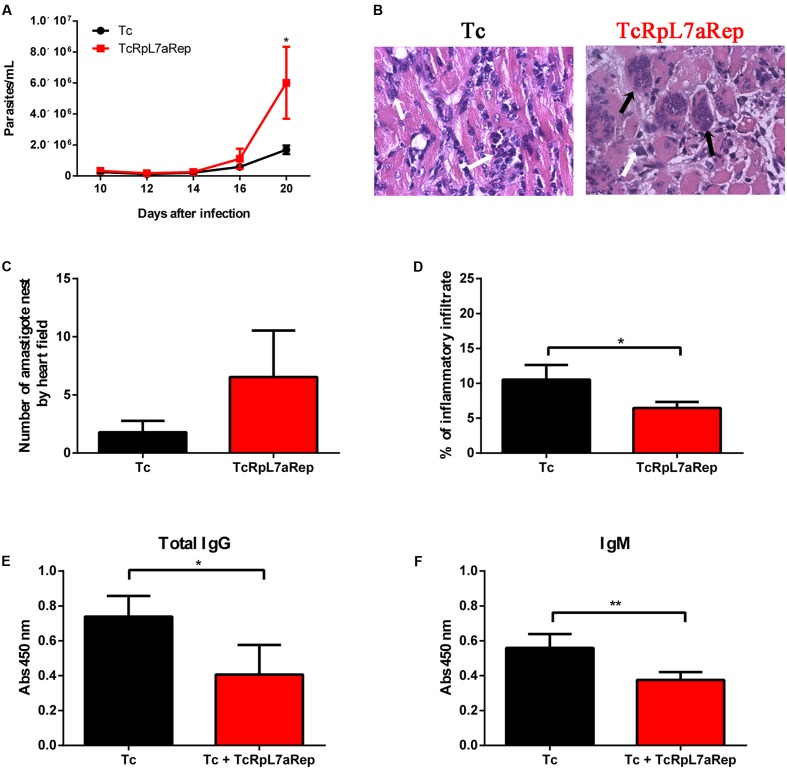
Immunization with TcRpL7aRep down-regulates immune response in *T. cruzi-*infected mice. Two groups of BALB/c mice, immunized three times with 10 μg of recombinant TcRpL7aRep protein plus CpG and alum or only with the two adjuvants with an interval of 14 days were challenged 3 weeks after the last immunization with 5,000 bloodstream trypomastigotes of the CL Brener strain. Parasitemia of *T. cruzi-*infected mice (Tc) or TcRpL7aRep-immunized and infected mice (Tc + TcRpL7aRep) was monitored starting on day 10 post-infection **(A)**. At the peak of parasitemia (day 20), mice were euthanized, their heart and serum samples were taken for histopathological and humoral analyses, respectively. Harvested hearts were fixed on paraformaldehyde, embedded in paraffin, then sectioned, and stained with H&E. Representative images of H&E stained heart sections (40×) are shown in **(B)**, the quantification of the numbers of intact amastigote nests is shown in **(C)**, and the percentages of inflammatory infiltrate calculated by determining the percentages of area on tissue sections containing inflammatory cells are shown in **(D)**. Data are reported as the mean ± SD of 20 microscopic fields (magnification = 40×) in one H&E stained heart section per animal. White and black arrows indicate inflammatory infiltrate and integral *T. cruzi* amastigote nest, respectively. Total IgG **(E)** and IgM **(F)** antibody levels were measured by ELISA using total *T. cruzi* extract (Tc extract) to coat the plates (^∗∗∗^*P* < 0.001; ^∗∗^*P* < 0.01; ^∗^*P* < 0.05).

To investigate the mechanisms responsible for the differences observed in the outcome of the infection when the group of infected naïve animals was compared with the group of animals that were immunized with TcRpL7aRep prior to infection, we analyzed the histopathology of heart tissues collected from both groups of animals at the peak of the parasitemia. As shown in **Figure 6A**, on day 20 post-infection, TcRpL7aRep immunized mice showed significantly higher parasitemia when compared to the control infected mice. Although histopathological analyses of heart tissues (**Figure 6B**) showed no significant difference in the numbers of amastigote nests (**Figure 6C**), a significant reduction in the inflammatory infiltrate was observed in the hearts of mice that were previously immunized with TcRpL7aRep compared to heart tissues from infected control mice (**Figure 6D**). Also in accordance with the increased parasitemia and mortality rates of animals that were immunized with the repetitive antigen before the infection, analyses of the humoral response against total parasite antigens showed that TcRpL7aRep-immunized mice produce lower levels of total IgG and IgM anti-*T. cruzi* antibodies compared to infected control animals that were not previously immunized with this antigen (**Figures 6E,F**). It is worth mentioning that re-stimulation of splenocytes collected from both groups of mice showed no differences regarding the production of IFN-γ and IL-10 (**Supplementary Figure S2**).

### Immunization with TcRpL7aRep Inhibits Humoral Response and Hypersensitivity Reaction against an Heterologous Antigen

**FIGURE 7:**
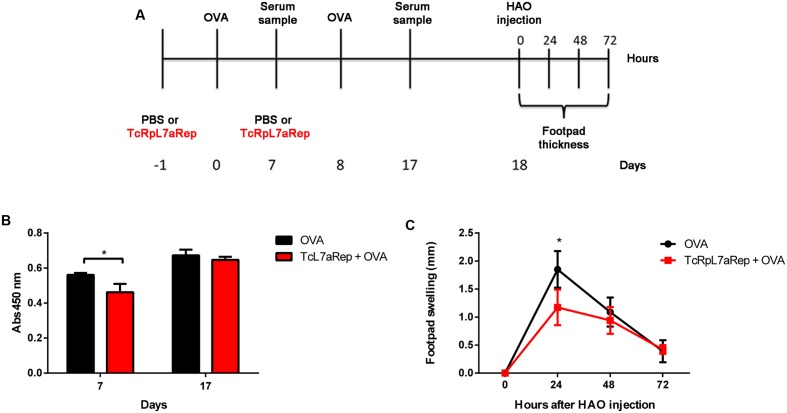
Immunization with TcRpL7aRep impairs humoral and hypersensitivity response against the heterologous antigen OVA. Two groups of BALB/c mice were immunized subcutaneously twice with an interval of 10 days, with 100 μg of OVA diluted in 50 μl of CFA. Animals from one group of mice were injected with 10 μg of TcRpL7aRep diluted in saline buffer, 24 h before each OVA immunization whereas the control group received saline injections. Serum samples were taken at days 7 and 17, and in day 18 both groups of mice were injected with 50 μl of 2% of HAO solution in one of the footpad. Footpad swelling was measured at 24, 48, and 72 h after HAO injection, as schematized on **(A)**. Anti-OVA IgG serum levels at days 7 and 17 were measured by ELISA **(B)**. Hypersensitivity response was determined by measuring footpad swelling and subtracting the footpad thickness after HAO injection minus the basal footpad thickness **(C)** (^∗∗∗^*P* < 0.001; ^∗∗^*P* < 0.01; ^∗^*P* < 0.05).

To further investigate the immune modulatory capacity of TcRpL7aRep we analyzed the response of TcRpL7aRep-immunized mice against an heterologous antigen like OVA. Two groups of BALB/c mice were immunized with two subcutaneous injections of OVA plus CFA adjuvant. As shown in **Figure 7A**, one group has been immunized with TcRpL7aRep diluted in buffered saline, 24 h before OVA immunization, whereas the other group received saline injections before OVA immunization. Similar to the antibody response against total parasite antigens, total IgG levels directed against OVA was significantly reduced on day 7 after OVA immunization in mice that were previously injected with TcRpL7aRep compared to control mice (**Figure 7B**). Consistent with the reduction in anti-OVA IgG present in the sera of TcRpL7aRep immunized mice, these animals presented reduced footpad inflammation 24 h after injection of HAO (**Figure 7C**). Thus, besides modulating the immune response against parasite antigens, these results indicate that TcRpL7aRep can also affect the humoral and cellular response against an antigen not present in the *T. cruzi* proteome.

### TcRpL7aRep Suppresses *in Vitro* B-Cell Proliferation

**FIGURE 8:**
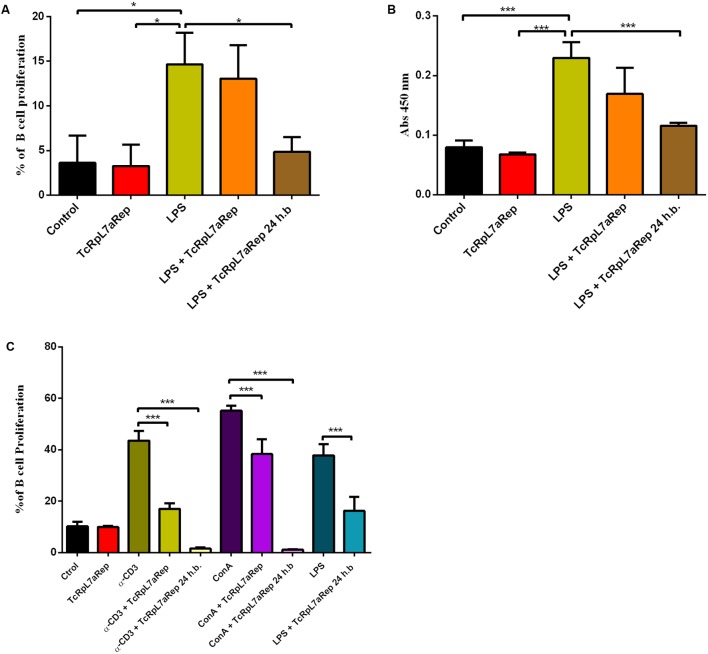
TcRpL7aRep inhibits B-cell proliferation and specific antibody synthesis. CFSE-labeled splenocytes from healthy BALB/c mice were incubated for 72 h with TcRpL7aRep at 10 μg/ml, LPS at 10 μg/ml (control group), or with LPS in the presence of TcRpL7aRep. The recombinant antigen was added together with the stimulus or 24 h before (24 h.b.). Complete RPMI medium was used as negative control. B-cell proliferation was measured by CFSE dilution of CD19+ gated cells by flow cytometry **(A)**. Total IgM levels on cell culture supernatants were measured by ELISA **(B)**. To analyze the effect of TcRpL7aRep on B cells submitted to different stimuli, CFSE-labeled SMC from BALB/c mice were incubated for 120 h with α-CD3, ConA, or LPS in the presence or absence of TcRpL7aRep at 10 μg/ml. The repetitive antigen was added together with the stimulus or 24 h.b. B-cell proliferation was measured by flow cytometry, as indicated by the dilution of the CFSE fluorescence of gated CD19+ cells compared to positive and negative controls **(C)** (^∗∗∗^*P* < 0.001; ^∗∗^*P* < 0.01; ^∗^*P* < 0.05).

Hypergammaglobulinemia, a condition that characterizes the acute phase of CD, is part of the non-specific immune response that contributes to delay the specific humoral response against the parasite (Minoprio et al., 1988; Bryan et al., 2010). Several *T. cruzi* proteins such as malate dehydrogenase (Montes et al., 2002), glutamate dehydrogenase (Montes et al., 2006), rTc24 (Cordeiro da Silva et al., 1998), and proline racemase (Bryan and Norris, 2010) have been identified as polyclonal B-cell activators during *T. cruzi* infection. Members of the TS multi-gene family containing the repetitive SAPA domain at its C-terminal region have also been identified as a strong polyclonal B-cell activator that induces a strong immune response during the acute phase of CD (Gao et al., 2002). To verify whether the repeat domain present in TcRpL7a could also act as a polyclonal activator of B cells, we incubated TcRpL7aRep with mouse splenocytes *in vitro* and determined B-cell proliferation rates by analyzing CFSE dilution of CD19+ gated cells by flow cytometry. Before performing flow cytometry, we analyzed the cytotoxicity of the antigen and showed that incubation of splenocytes with 10 μg/ml of the TcRpL7aRep antigen does not significantly affect cell viability (**Supplementary Figure S3**). As shown in **Figure 8A**, in contrast to LPS, a well know polyclonal activator, incubation with TcRpL7aRep does not cause B-cell proliferation and does not induce antibody production. Interestingly, when splenocytes were incubated for 24 h with TcRpL7aRep before LPS stimulation, we observed a 60% inhibition of B-cell proliferation (**Figure 8A**) and a 50% inhibition of IgM antibody production induced by LPS (**Figure 8B**). Statistically significant differences in cell proliferation and antibody production were only observed when splenocytes were incubated with TcRpL7aRep 24 h prior the addition of LPS, suggesting that the two molecules may compete for their cell surface counterparts (**Figures 8A,B**).

To verify whether the immunosuppressive effect due to the presence of TcRpL7aRep could also be observed if B cells were activated through different mechanisms, mouse splenocytes were stimulated with α-CD3, ConA, or LPS in the presence or absence of TcRpL7aRep. As expected, α-CD3, ConA, and LPS strongly induces B-cell proliferation (**Figure 8C**). Similar to the results observed with LPS stimulation, incubation of splenocytes with TcRpL7aRep 24 h before the addition of α-CD3 and ConA totally abrogated B-cell proliferation (**Figure 8C**). Distinct from the results observed with LPS stimulation, incubation of B cells with α-CD3 and ConA resulted in a significant inhibition of proliferation if TcRpL7aRep was added at the same time of the addition of the mitogenic stimuli (**Figure 8C**). Taken together, these results indicate that TcRpL7aRep induces an anergic state of B cells, which results in a decreased capacity to proliferate in response to different stimuli or in response to parasite infection.

## Discussion

*Trypanosoma cruzi* infection elicits a complex innate and adaptive host immune response, which controls the parasite burden but often does not eradicates it. One of the biggest challenges faced by research groups studying CD is to understand the mechanisms responsible for the dynamic equilibrium established between this parasite and its host. Since there is no effective treatment for chronic infections and, despite significant advances in vector control, new infections continue to occur, the elucidation of immune evasion mechanisms elicited by this parasite remains as the main prerequisite for the rational development of a vaccine against CD.

Aiming at identifying *T. cruzi* antigens that are targets of the host immune response, we and others have isolated several antigens using cDNA libraries constructed with cDNA derived from distinct parasite forms and sera from chronic CD patients (DaRocha et al., 2002; Goto et al., 2008). In these studies, it became evident that proteins containing repeat domains are immunodominant antigens that elicit a strong host humoral response. Among the *T. cruzi* antigens containing repetitive domains, we identified a group of ribosomal proteins, one of them, the homolog of the eukaryotic ribosomal protein L7a that is been used as target for sorodiagnostics. Similar studies with *Leishmania* spp., *Plasmodium falciparum*, *Neospora caninum*, and *Toxoplasma gondii*, have also shown that ribosomal proteins and proteins containing repeat domain are important targets of the immune response (Soto et al., 1995). Such strong immune response against repeat domains from various antigens lead to the “smoke screen” hypothesis, i.e., that the response against repeat domains present in parasite antigens is part of an immune-evasion mechanism that diverts the immune response away from more important parasite targets (Kemp et al., 1987). Alternatively, repetitive domains such as the SAPA domain present in the *T. cruzi* TS, which cause polyclonal activation of B cells results in depletion and/or clonal exhaustion of B cells and weakening the specific humoral host immune response (Minoprio et al., 1988; Gao et al., 2002; Bryan et al., 2010).

We have previously shown that the *T. cruzi* ribosomal protein TcRpL7a contains a repeat domain that is strongly recognized by antibodies from chronic CD patients (Pais et al., 2008). By immunizing mice with full-length protein as well as with truncated versions of the protein before challenging with *T. cruzi* trypomastigotes, we found evidence indicating that the repeat domain of TcRpL7a is part of the strategic arsenal developed by *T. cruzi* to down-modulate the host immune response and help to establish a long lived infection in the mammalian host. Similar to patients chronically infected with *T. cruzi*, mice immunized with the full-length protein produce high levels of IgG antibodies against this antigen as well as significant levels of IFN-γ and IL-10 produced by splenocytes that were re-stimulated with the full-length TcRpL7a. Mice immunized with the truncated version containing only the non-repetitive domain, where the major CD4 and CD8 epitopes are located, also produce IgG and IFN-γ, albeit at significantly low levels compared to mice immunized with TcRpL7a. Consistently, the two groups of mice that were immunized with the full-length (TcRpL7a) or with TcRpL7aΔRep were partially protected against a challenge with trypomastigotes. These results are similar to a number of studies that correlate protection levels and antibody, IFN-γ, and IL-10 production by immunized animals (Plata et al., 1984; Gazzinelli et al., 1992; Nickell et al., 1993; Junqueira et al., 2010; Roffê et al., 2012). These results are also in agreement with the presence of CD4 and CD8 epitopes in the non-repetitive domain, i.e., present in the full-length TcRpL7a and TcRpL7aΔRep recombinant proteins. In contrast, mice immunized with the truncated version containing only the repeat domain (TcRpL7aRep) have a completely different outcome, with increased parasitemia and 100% mortality compared to all other groups. It is noteworthy that although this region of the protein contains all B-cell epitopes, but lacks major CD4 or CD8 T cells epitopes, immunization with TcRpL7aRep did not result in the production of either IgG or IgM antibodies. Thus, the lack of IgM production in mice immunized with TcRpL7aRep suggests that the repeat domain has an immunomodulatory effect over B cells. Furthermore, besides having a decreased humoral response against parasite antigens, the group of mice immunized with TcRpL7aRep showed reduced inflammatory infiltrate in heart tissues even when the animals were analyzed at the pick of parasitemia.

Although the absence of CD4 and CD8 T cells epitopes in the repeat domain of TcRpL7a explains the lack of TcRpL7a-specific IFN-γ, IL-10, and antibody production in mice immunized with TcRpL7aRep, the lack of epitopes may not be the only factor responsible for the increased susceptibility to a challenging infection in animals immunized with TcRpL7aRep. It is noteworthy that cytokine production against whole parasite extract was not affected by this immunization. The results showing decreased levels of anti-*T. cruzi* IgG and IgM antibodies and a lower percentage of inflammatory infiltrate in the heart of these animals led us to hypothesize that immunization with TcRpL7aRep results in an immunosuppressive state, a hypothesis that was corroborated by the results of immunization experiments with the OVA antigen. Since immunization with a non-related antigen also resulted in reduced humoral and hypersensitive response against the OVA antigen in animals that were previously immunized with TcRpL7aRep compared to controls, we speculate that the repeat domain of TcRpL7a acts as an immunosuppressive factor that downmodulates the host immune response in a nonspecific manner.

Based on previous studies showing that the repeat SAPA domain present in a sub-group of TSs acts as a polyclonal B-cell activator (Gao et al., 2002; Bryan et al., 2010), we performed *in vitro* proliferation assays to investigate the immunomodulatory mechanism associated with TcRpL7aRep immunization. Surprisingly, instead of acting as a polyclonal B-cell activator, the TcRpL7a repeat domain inhibited B-cell proliferation *in vitro*. As shown by *in vitro* proliferation assays of splenocytes taken from healthy mice submitted to different stimuli, incubation of these cells with TcRpL7aRep resulted in an anergic state of B cells, i.e., they became less capable to proliferate or to secrete antibodies after stimulation in response to different polyclonal activators. A possible mechanism to explain the immunosuppressive effect of TcRpL7a repeat domain is based on the immunon model, originally described by Dintzis et al. (1976). According to this model, multivalent and repetitive antigens can activate B cells without the involvement of T helper cells if the repeat domain is able to simultaneously crosslink more than 20 B-cell receptors (BCRs) (Dintzis et al., 1976, 1983). If the repeat domain contains less than 20 B-cell epitopes, which is below the threshold required for induce proliferation, a tolerance response takes place (Dintzis et al., 1983; Sulzer and Perelson, 1997). This principle was used to design drugs such as LJP-394 (abetimus sodium), comprised of four double-stranded oligodeoxyribonucleotide epitopes that induce tolerance in B cells by cross-linking surface antibodies and which has been tested as a treatment for systemic lupus erythematosus (Mosca et al., 2007). Since the repeat domain of TcRpL7a has less than 10 epitopes, we proposed that it acts as a type II T-cell independent antigen that induces B-cell tolerance. Thus, the results showing that B cells that were pre-incubated *in vitro* with TcRpL7aRep fail to proliferate or to secrete antibodies in response to α-CD3, ConA, or LPS may be due to the binding of the repeated antigen to a small number of BCR molecules. During the infection *in vivo*, this model not just explains the lack of antibody production after TcRpL7aRep immunization but also the down modulation of the immune response against parasite antigens as well as against OVA and, most importantly the increased susceptibility of infected animals that were previously immunized with TcRpL7aRep. It is likely that such significant tolerogenic effect may not result from crosslinking BCRs in one specific B-cell clone, but instead the repeat domain of TcRpL7a must be able to crosslink BCRs from several B-cell clones, or alternatively, it binds to innate like B cells, such as marginal zone B cells or B1 cells, which possess more conserved and less variable BCR (Casali and Schettino, 1996; Panda and Ding, 2015). Consistent with a hypothesis based on the tolerogen/immunon model, the difference in the protection capacity observed between the full-length TcRpL7a and the truncated version that does not carry the repeat domain may be due to the fact that immunization with the full-length antigen induces antibody production against the repeat domain TcRpL7aRep, which may prevent the binding of this region of the protein to BCRs.

How a parasite antigen that is part of the ribosome can exert this effect over host B cells is a question that has been raised since the early studies showing antibody response against conserved parasite antigens that are part of multi-component complexes (Requena et al., 2000). As part of a ribonucleoprotein complex, it can be expected that TcRpL7a might be an abundant circulating antigen that is released after parasite lysis. In light of new findings showing that microvesicles play a key role in host–parasite interaction (Ramirez et al., 2017), and that several parasite antigens including the repeat domain (N-terminal region) of MASP proteins are released by *T. cruzi* exovesicles (Lozano et al., 2017), we can also considered the possibility that TcRpL7a may be actively released from exovesicles that are shedded by bloodstream trypomastigotes. It is worth noting that proteomic analyses showed the presence of ribosomal proteins in exovesicles released by *T. cruzi* trypomastigotes (Bayer-Santos et al., 2013). It is also possible that the increased protein stability conferred by the repeat domain present in TcRpL7a, either released after parasite lysis or through exovesicles formation, contributes to sustaining the immune modulatory effect of this antigen and helps promote the immunological balance that is established between host and parasite during *T. cruzi* infection.

## Author Contributions

Conceived and designed the experiments: CTA, BV, GB-C, ER, and ST. Performed the experiments: CTA, BV, GB-C, BG-F, CJ, REA, and ER. Analyzed the data: CTA, BV, GB-C, BG-F, CJ, REA, RG, HdCS, ST, and ER. Contributed reagents/materials/analysis tools: CJ, RG, and ST. Wrote the paper: CTA, BV, ER, HdCS, and ST.

## Conflict of Interest Statement

The authors declare that the research was conducted in the absence of any commercial or financial relationships that could be construed as a potential conflict of interest. The handling Editor declared a shared affiliation, though no other collaboration, with the authors ST, REA, RG, amd HdCS.

## Footnotes

1 www.who.int/chagas/disease/en/

2 www.tritrypdb.org

## Supplementary Material

**FIGURE S1 |** Prediction of linear B- and T-cell epitopes. Linear epitopes for B cells **(A)** and T cells MHC class I **(B)** and MHC class II **(C)** were predicted from the amino acid sequence of TcRpL7a derived from the CL Brener strain using the software BepiPred and IEDB are underlined. The repeat domain is highlighted in bold. Scores obtained for each epitope are shown by the numbers below the lines.

**FIGURE S2 |** Cellular response in TcRpL7aRep-immunized *T. cruzi-*infected mice is not affected. *T. cruzi-*infected mice (Tc) or TcRpL7aRep-immunized and infected mice (Tc + TcRpL7aRep) were sacrificed at the peak of the parasitemia, and the splenocytes were cultured for 72 h in RPMI medium as negative control, or in presence of 10 μg/ml Tc extract. Levels of IFN-γ **(A)** and IL-10 **(B)** in culture supernatants were measured by ELISA (^∗∗∗^*P* < 0.001; ^∗∗^*P* < 0.01; ^∗^*P* < 0.05). No statistically significant differences in IFNg and IL-10 production by cells from infected mice that were previosuly immunized with TcRpL7a (Tc + TcRpL7aRep) compared to cells from mice that were only infected with *T. cruzi* (Tc) (IFNg, *P*-value = 0.8000; IL-10 *P*-value = 0.1000, Mann–Whitney test) were found.

**FIGURE S3 |** TcRpL7aRep cytotoxicity. Splenocytes from healthy BALB/c mice were incubated for 144 h with complete RPMI medium (Control) alone or in the presence of TcRpL7aRep at 10, 5, or 1 μg/ml. Cytotoxicity assays were performed by incubating cells for 2 h at 37°C with 80 μl of culture medium plus 20 μl of PBS 1× solution with 2.5 mg/ml 3-(4, 5-dimethylthiazolyl-2)-2,5-diphenyltetrazolium bromide (MTT) reagent. After solubilization of the formazan cristal with 10% SDS, absorbance was measured at 595 nm (^∗∗∗^*P* < 0.001; ^∗∗^*P* < 0.01; ^∗^*P* < 0.05).

**TABLE S1 |** T-cell and B-cell epitope predictions for the TcRpL7a antigen.
