# Supplementary figures and images for: Down Modulation of Host Immune Response by Amino Acid Repeats Present in a Trypanosoma cruzi Ribosomal Antigen
Source: Front Microbiol. 2017 Nov 10;8:2188. doi: 10.3389/fmicb.2017.02188 (PMC5686100; doi:10.3389/fmicb.2017.02188)

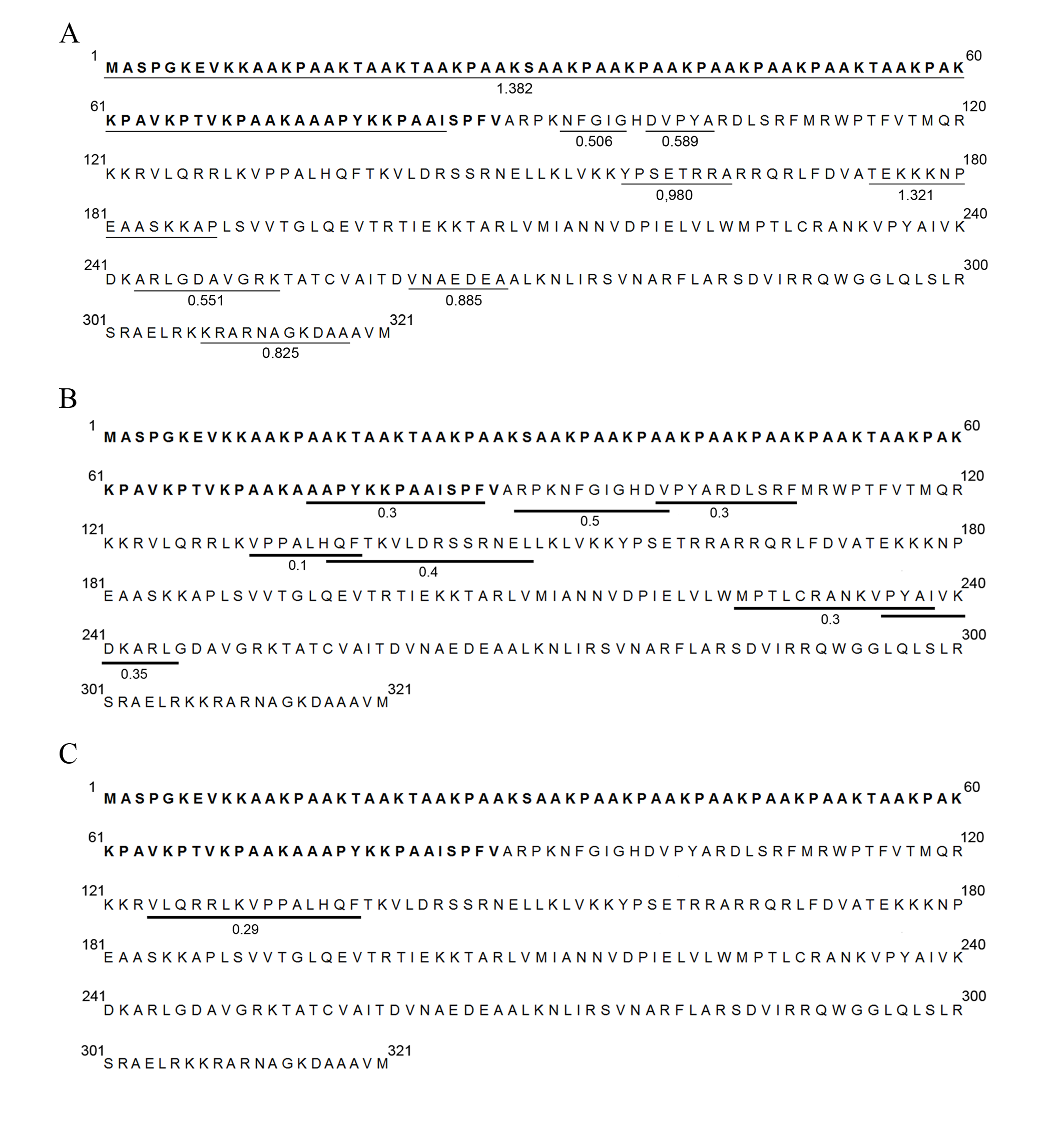

Supplement: Supplementary file 1 [file Image_1.TIF]

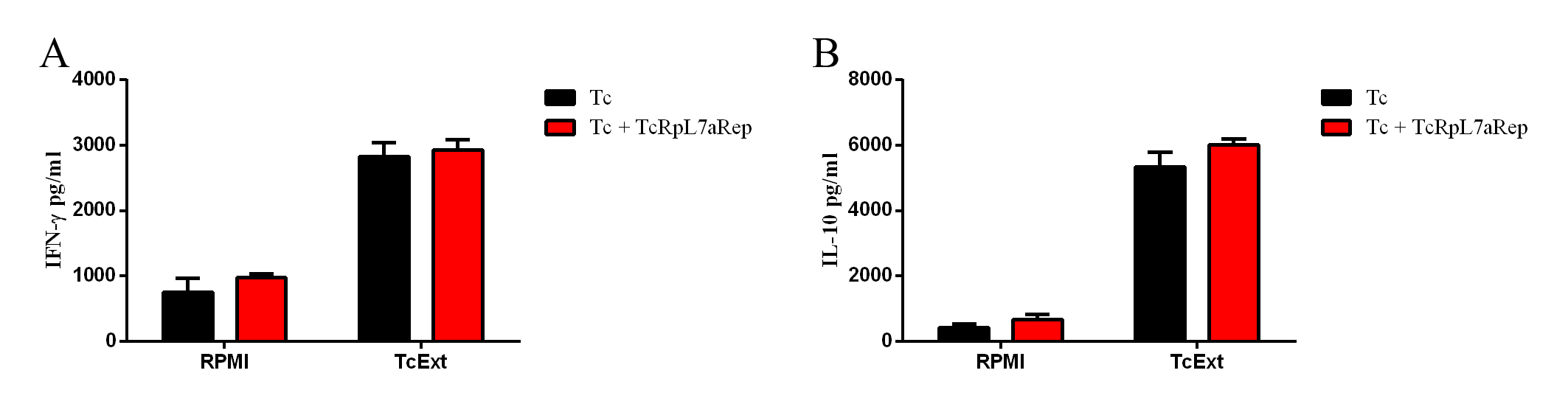

Supplement: Supplementary file 2 [file Image_2.TIF]

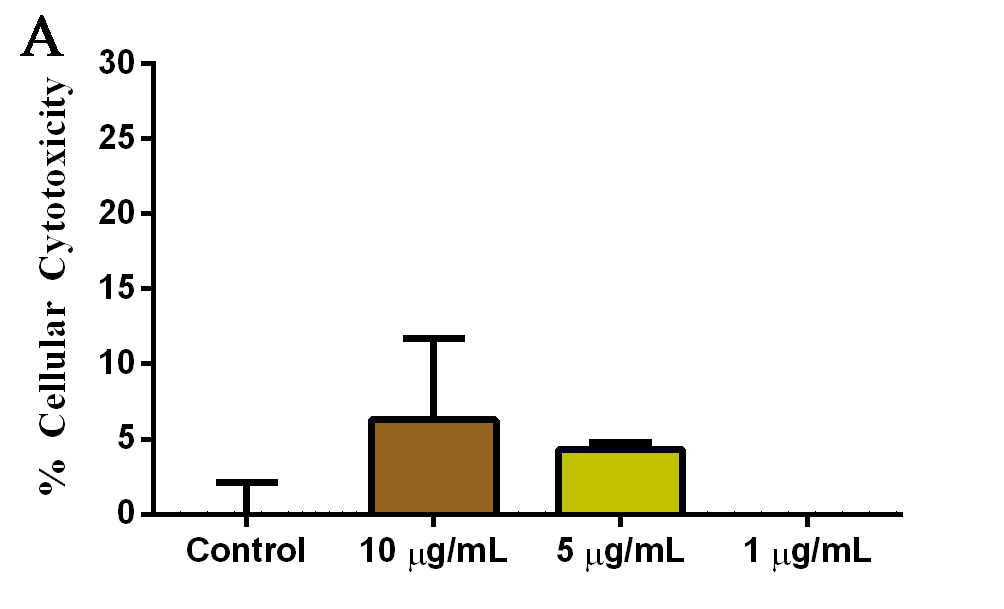

Supplement: Supplementary file 3 [file Image_3.TIF]
