# Supplementary material for: Down Modulation of Host Immune Response by Amino Acid Repeats Present in a Trypanosoma cruzi Ribosomal Antigen
Source: Front Microbiol. 2017 Nov 10;8:2188. doi: 10.3389/fmicb.2017.02188 (PMC5686100; doi:10.3389/fmicb.2017.02188)
Supplement: Supplementary file 4 [file Table_1.PDF]

Epitope predictions

B cells				
Start	End	Peptide	Score	
1	84	MASP…PAAI	1.382	
93	97	NFGIG	0.506	
99	103	DVPYA	0.589	
157	164	YPSETRRA	0.980	
174	188	TEKKKNPEAASKKAP	1.321	
243	252	ARLGDAVGRK	0.551	
262	268	VNAEDEA	0.885	
308	318	KRARNAGKDAA	0.825	
MHC I				
Allele	Start	End	Peptide	Percentile rank
Ld > Dd	125	132	VPPALHQF	0.1
Ld > Dd	219	232	MPTLCRANKVPYAI	0.3
Ld > Dd	94	103	VPYARDLSRF	0.3
Dd >> Ld = Kd	75	87	AAPYKKPAAISPF	0.3
Kd	235	245	PYAIVKDKARL	0.35
Kd	131	144	QFTKVLDRSSRNEL	0.4
Ld	84	94	RPKNFGIGHDV	0.5
MHC II				
Allele	Start	End	Peptide	Percentile rank
H2-IAd	118	132	VLQRRLKVPPALHQF	0.29
